# The Effect of Thermal Oxidation on the Photothermal Conversion Property of Tantalum Coatings

**DOI:** 10.3390/ma14144031

**Published:** 2021-07-19

**Authors:** Ding Ding, Qingping Zeng, Feng He, Zhuojun Chen

**Affiliations:** 1 College of Materials Science and Engineering, Hunan University, Changsha 410082, China; dingding@cs48.com; 2 Key Laboratory of Thin Film Sensing Technology for National Defense of Hunan Province, 48th Research Institute of China Electronics Technology Group Corporation, Changsha 410111, China; zengqp@cs48.com (Q.Z.); hefeng@cs48.com (F.H.); 3 School of Physic and Microelectronics, Hunan University, Changsha 410082, China

**Keywords:** tantalum oxides, thermal oxidation, photothermal conversion, oxygen vacancy

## Abstract

In this study, tantalum coatings are deposited by a plasma spraying method aiming at enhancing the biocompatibility of the titanium implant. Tantalum oxide coatings are gained through the thermal oxidation of tantalum coatings at different temperatures for photothermal therapy. The effect of thermal oxidation on the morphology, composition, and structure of tantalum coatings has been studied. The UV–VIS–NIR spectra results, cancer therapy effect in vitro, and photothermal conversion properties among the tantalum oxide coatings under varied thermal treatment conditions are compared comprehensively. It has been proven that the tantalum coating treated at 200 °C exhibits the most intense NIR adsorption, the highest photothermal conversion effect, and the most excellent photothermal ablation effect in vitro. The results reveal that incomplete oxidation at a low temperature leads to the formation of oxygen vacancies, which narrow the band gap; this promotes its photothermal conversion ability.

## 1. Introduction

Tantalum (Ta) and its oxides are gaining increasing interest for their excellent biocompatibility [1,2,3]. Plasma-sprayed tantalum coating with a high bonding strength and a porous surface structure is suitable for metal implants, as the biological performance can be improved by combining the substrate with such biocompatible and corrosion-resistant surfaces [4,5]. Metal implants of titanium and its alloy have been used extensively for hard tissue disorder treatments, owing to their excellent mechanical properties and biocompatibility [6]. Some studies have demonstrated that plasma-sprayed tantalum coating can enhance the osteogenic properties of the titanium implant in the early stage [7,8,9].

Currently, the photothermal conversion property of biocompatible materials is considered a key parameter for photothermal therapy in bone cancer post-surgery [10,11]. Photothermal conversion means converting the absorbed photon energy from optical illumination into heat energy, which makes it a promising method of therapy to kill cancer cells through the generated heat. Therefore, it is particularly important to develop a bifunctional coating with properties of bone defect regeneration and photothermal conversion for killing the remaining tumor cells.

This photothermal conversion property is critical for photothermal therapy in bone cancer post-surgery. Furthermore, photothermal agents with enhanced visible and near-infrared (NIR) light absorption exhibit a higher photothermal conversion efficiency, which is expected to cure bone cancer effectively [12]. In recent years, black oxidation materials such as hydrogenated-black TiO_2_ and black plasma-sprayed tantalum oxide coatings have been explored for their thermal conversion efficiency [13,14]. Black TiO_2_ and Ta_2_O_5_ have been demonstrated to possess an enhanced NIR absorption and visible light absorption that are mainly attributed to the existence of oxygen vacancies.

In metal oxides, the major structure defect is oxygen vacancy, which directly leads to the narrowed band gap and a resultant increase in photo absorption. Consequently, the photothermal conversion efficiency in the visible–NIR region can be enhanced [15]. Ta can become oxidized into various oxidation states such as pentoxide and many other suboxide states. The oxidation states of Ta through the diffusion of oxygen depend on different thermal treatment temperatures [16]. According to different oxidation treatment conditions, the treated tantalum coatings consist of different phase compositions, including fully oxidized Ta_2_O_5_, single-phase tantalum, and tantalum suboxides [17,18]. To note, the formation of suboxides is usually accompanied by oxygen deficiency, which can promote the photothermal efficiency of tantalum coatings.

In this study, plasma-sprayed tantalum coatings after thermal oxidation at different temperatures have been prepared and the effect of thermal oxidation on the photothermal conversion property has been analyzed. Our study aims to explore a way to achieve a bifunctional surface modification layer that can be used post-surgery for bone cancer photothermal therapy.

## 2. Materials and Methods

### 2.1. Preparation of Plasma Sputtered Tantalum Coatings

Commercially available pure tantalum powder (purity 99.99%, particle size of about 70 μm,) and titanium plates (purity 99.9%) (both from Zhongsheng Hengan New Material Technology Co., Ltd. Beijing, China) were used in the preparing work. A vacuum plasma spraying (VPS) system (Sulzer Metco, Winterthur, Switzerland) was used to fabricate the tantalum coating. Ta coatings with a thickness of 300 μm were sprayed on titanium plates with a dimension of Φ 10 mm × 2 mm. The spraying power was 40 kW at a spray distance of 280 mm, under a plasma Ar and H_2_ gases of 40 and 10 slpm, respectively. The powder feed rate was 24 g/min.

The vacuum-sprayed tantalum coatings were thermally treated in an ambient atmosphere at different temperatures (200 °C, 400 °C, and 600 °C) for 1 h. Thermal treatments were handled in the tubular furnace closed with double insulation plugs. Ta coatings treated at different temperatures were obtained and referred to as VT, VT200, VT400, and VT600, respectively.

### 2.2. Characterization and Photothermal Test

The phase identification of VT, VT200, VT400, and VT600 was performed using XRD (D8 Discover, Bruker AXS Gmbh, Karlsruhe, Germany) over a 2Theta range, from 20° to 80° operating at 45 kV and 30 mA. The XRD data analysis and phase identification were done with Jade software. The surface morphologies of the VT, VT200, VT400, and VT600 coatings were characterized by SEM (Hitachi S-4800, Tokyo, Japan).

Electron spin resonance (ESR; JEOL, FA200), and photoelectron spectroscopy (XPS; ThermoFisher Scientific Escalab 250 spectrometer, Waltham, MA, USA) were also carried out.

The electrochemical properties were tested using a CHI166E electrochemical workstation (CH Instrument Inc., Shanghai, China) in a standard three-electrode cell of 300 mL at 37 °C. VT, VT200, VT400, and VT600 were used as the working electrodes. A quadrate platinum slice with a size of 15 mm × 15 mm × 2 mm was used as the counter electrode. A saturated calomel electrode (SCE) was used as the reference electrode. The polarization curves of these films were measured at a scan rate of 0.01 mV/s. The tests were carried out in simulated body fluid (SBF).

To compare the photothermal conversion performances of different tantalum coatings, a multimode pump laser with a wavelength of 808 nm with a high power (Connect Fiber Optics Co., Shanghai, China) was employed to directly irradiate the coatings contained in the plastic tube. Then, the temperatures of the coatings were recorded.

### 2.3. Photothermal Ablation In Vitro

MG-63 cells (Sciencell, Carsbad, CA, USA) were seeded into 48-well culture plates at a density of 1 × 10^4^ cells per well in fresh DMEM at 37 °C in 5% CO_2_. These cells were then irradiated for various times (0, 10, and 20 min) with an 808 nm laser at power densities of 0.5 W cm^−2^. Then, a standard CCK-8 assay was used to evaluate the viability of cells (*n* = 5). The photothermal effect of the coatings was verified by confocal laser scanning microscopy (CLSM) images acquired using a FV1000 microscope (Olympus Co., Tokyo, Japan).

## 3. Results and Discussion

### 3.1. Surface Characterization

**Figure 1:**
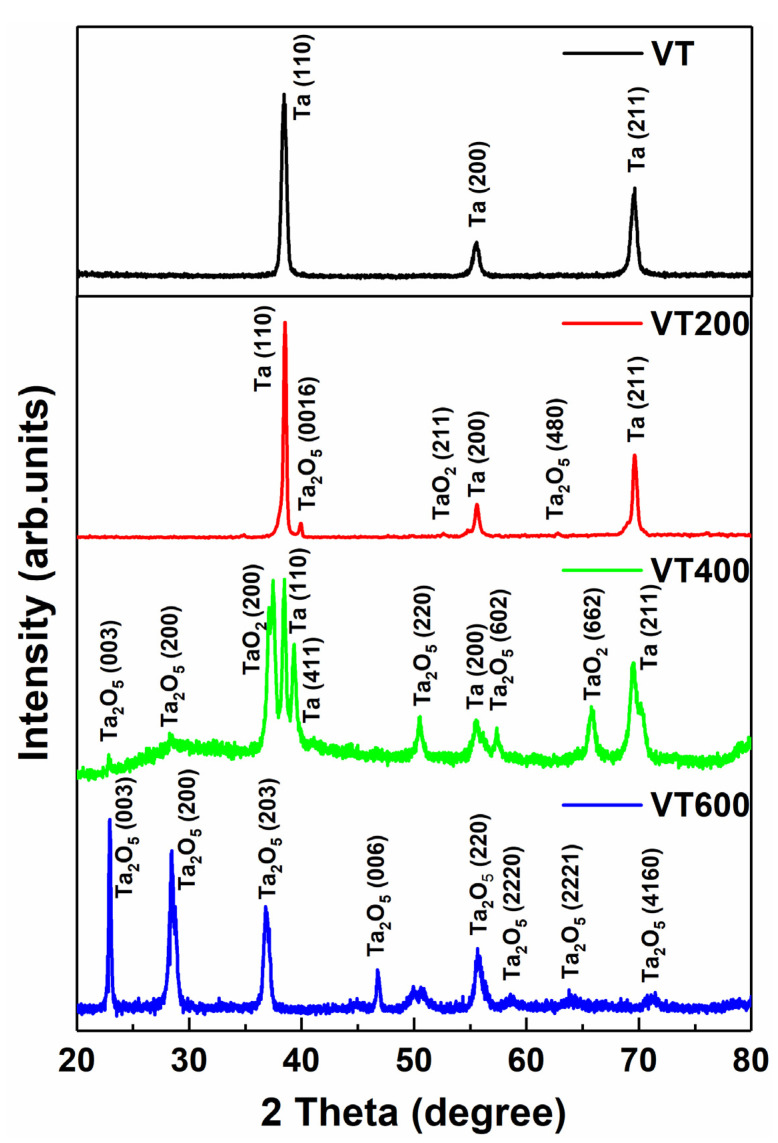
The XRD diffraction patterns of the surface of tantalum coatings thermal treated at different temperatures.

Figure 1 shows the XRD pattern of Ta coatings under different thermal treatment conditions for 1 h. It can be observed that the untreated tantalum coating (VT) contains only the single-phase Ta, whose diffraction peaks mainly come from 2Theta = 38.47°, 55.55°, and 69.58°, corresponding to (110), (200), and (211) planes of a cubic structure, respectively. After thermal treatment of 200 °C and 400 °C, the Ta coatings contain multi-phases of tantalum oxide, which are a mixture of Ta_2_O_5_ and TaO_2_ [19]. The single Ta phase is still present as the coatings are incompletely oxidized in a sealed heating furnace at 200 °C and 400 °C. When the thermal treatment temperature increases to 600 °C, the VT600 coating is completely oxidized to Ta_2_O_5_, which is a mixture of orthorhombic *β*−Ta_2_O_5_ and hexagonal *δ*−Ta_2_O_5_.

**Figure 2:**
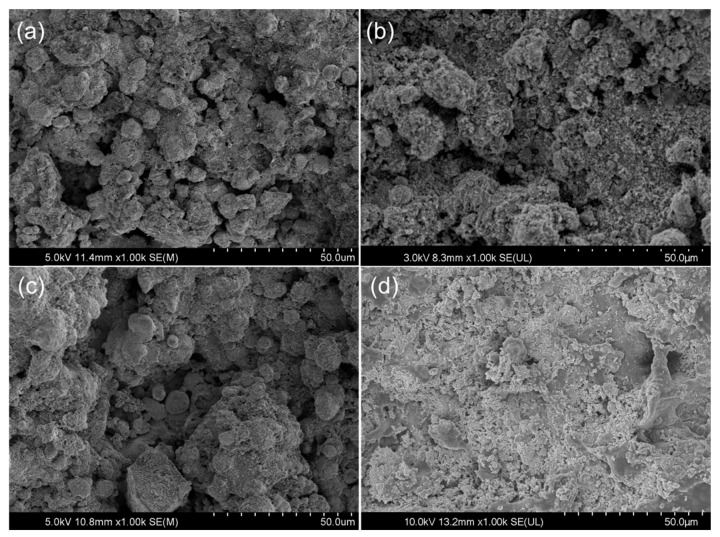
SEM images of (**a**) as-sprayed tantalum coating surface and surfaces tantalum coatings thermal treated at: (**b**) 200 °C, (**c**) 400 °C, and (**d**) 600 °C.

Figure 2 shows the surface morphology of the Ta coatings under different thermal oxidation conditions. Under the conditions of 200 °C and 400 °C heat treatments, there is no significant difference in the surface morphology of coatings, with both exhibiting rough porous structures similar to those without the heat treatment. When the heat treatment temperature reaches 600 °C, a complete thermal oxidation emerges and the porous structure of the coating disappears. It can be seen from the image that the coating under the highest temperature experiences pulverization and peeling due to the increase in temperature. Powder particles appear throughout the coating. Combined with the analysis of the XRD pattern of VT600, the complete oxidation coating, which could undergo a polymorphic transformation during thermal treatment at a high temperature, can result in spallation, owing to the stress generated by the phase transformation [20].

**Figure 3:**
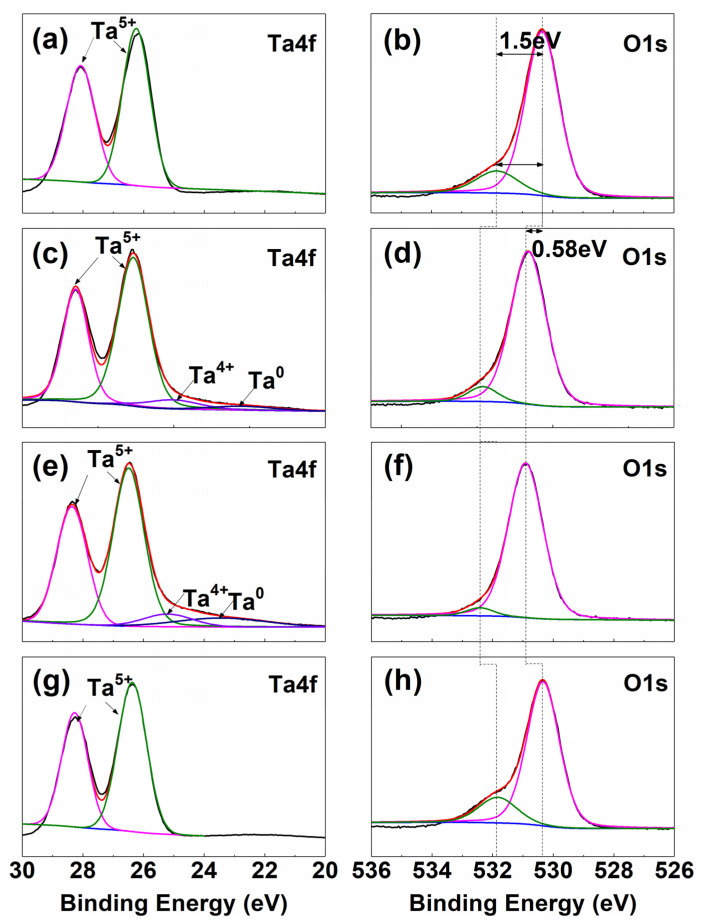
XPS survey spectra of Ta 4f core-level lines for (**a**) VT, (**c**) VT200, (**e**) VT400, and (**g**) VT600. XPS spectra of the Ta 4f and O 1s core-level lines for (**b**) VT, (**d**) VT200, (**f**) VT400, and (**h**) VT600.

Figure 3 presents the high-resolution XPS Ta 4f spectra and O1s spectra of the Ta coatings. As shown in Figure 3a,c,e,g, the high-resolution XPS Ta 4f spectra can be deconvoluted into multiple peaks. The contribution of Ta^5+^, which comes from tantalum pentoxide, is clearly observed in all four spectra, with the Ta 4f_7/2_ and Ta 4f_5/2_ peaks at binding energies of 26.0 eV and 27.9 eV, respectively [21,22]. After thermal treatment at 200 °C and 400 °C, the Ta 4f spectra is separated into four sets of peaks representing the different states of the oxides and metals. Beside the peaks at Ta 4f_7/2_ and Ta 4f_5/2_, the Ta 1f specta also displays two peaks at energies of 25.2 eV and 23.4 eV, in accordance with the states of Ta^4+^ and metallic Ta [23,24]. The existence of the Ta suboxide TaO_2_ in the film is confirmed by the peak between Ta^5+^ and metallic Ta [25]. Hence, the thermal treatment of the Ta coating at 200 °C and 400 °C can generate surface layers composed of Ta_2_O_5_, Ta suboxide, and Ta, which is perfectly in line with the results of the XRD patterns. It can be seen from Figure 3g that the XPS line drift of VT600 is close to that of Ta_2_O_5_.

Figure 3b,d,f,h shows the high-resolution XPS O spectra. In Figure 3b, it can be seen that the peak positions of the O1s spectra of VT200 and VT400 are 0.58 eV higher than that of Ta_2_O_5_, which is consistent with the oxygen vacancy ionization energy measured by Mikhaelashvili et al. [26]. The shift of the O1s spectra is due to the formation of oxygen vacancy, which leads to the introduction of additional localized states in the band, thus changing the Fermi level [27,28]. It can be seen from Figure 3h that the XPS line drift of VT600 recovered in the same way as that in Figure 3b. This is because when the thermal treatment temperature increases to 600 °C, VT600 has been fully oxidized, and there is no oxygen vacancy in the coating, with all of them being +5 valence Ta.

**Figure 4:**
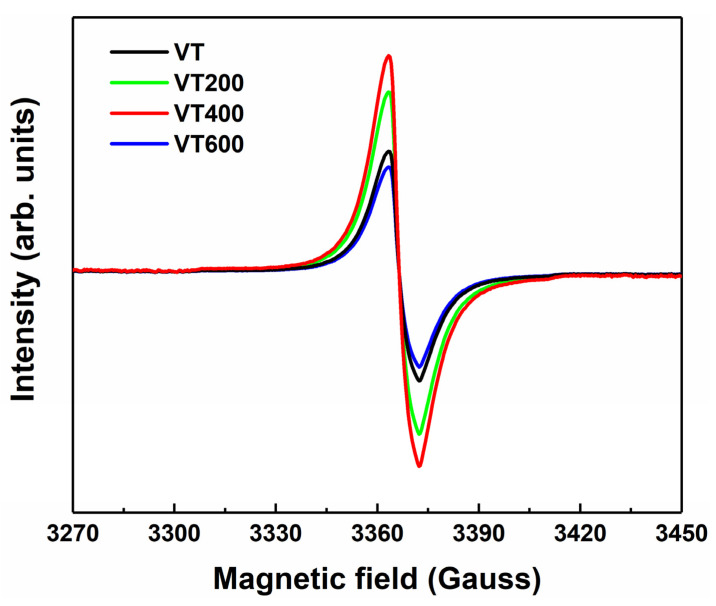
ESR spectra of VT, VT200, VT400, and VT600.

To further study the oxygen vacancy defects in the tantalum coating samples, ESR tests were carried out. Figure 4 shows the spectra of the coatings thermally treated at different temperatures. VT200 and VT400 have stronger ESR signals, which are attributed to oxygen vacancies [29,30]. The ESR signals of VT and VT600 are relatively weakened compared with VT200 and VT400, indicating decreased concentrations of the oxygen vacancies.

**Figure 5:**
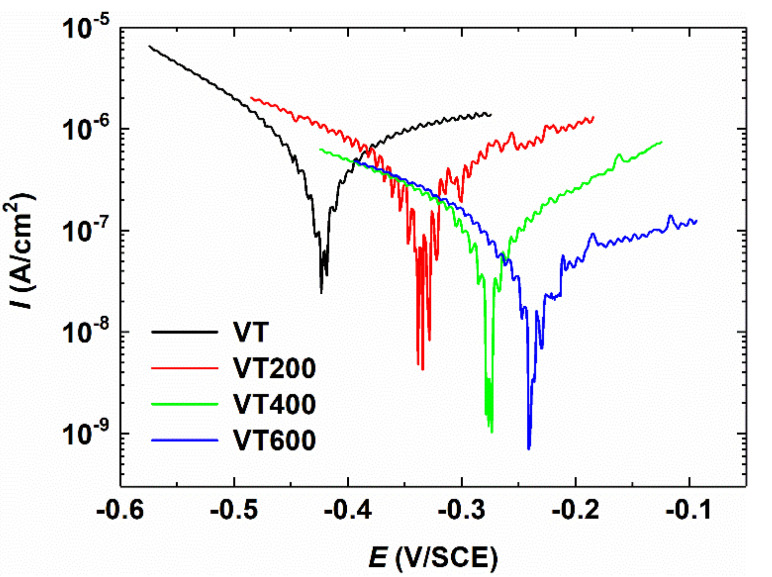
Polarization plots of tantalum coatings thermal treated at different temperatures.

For biomaterial implants, being biologically innocuous is expected. Thus, corrosion resistance is also significant, which is responsible for the chemical stability. Figure 5 shows the polarization curves of the tantalum coatings thermally treated at different temperatures. The electrochemical results indicate that the untreated VT sample has a relatively smaller Ψ_re_ of −0.42 V (vs. SCE) and I_o_ of 3.5 × 10^−7^ A/cm^2^, which indicates a lower resistance ability against the charge transfer at the layer/substrate interface. After thermal treatment for 1 h, the Ta coating samples are initially covered by inert Ta_2_O_5_ and Ta suboxides. Thus, the VT200 and VT400 samples exhibit smaller I_corr_ of 1.8 × 10^−7^ A/cm^2^ and 5.0 × 10^−8^ A/cm^2^, respectively. The smaller the corrosion current density, the lower the corrosion rate [31]. It is proven that the thermal treatment can enhance the corrosion resistance of the Ta coating. The Ta coating sample treated at 600 °C achieves the lowest I_o_ of 2.2 × 10^−8^ A/cm^2^. 

### 3.2. Photothermal Effect of Tantalum Coatings

**Figure 6:**
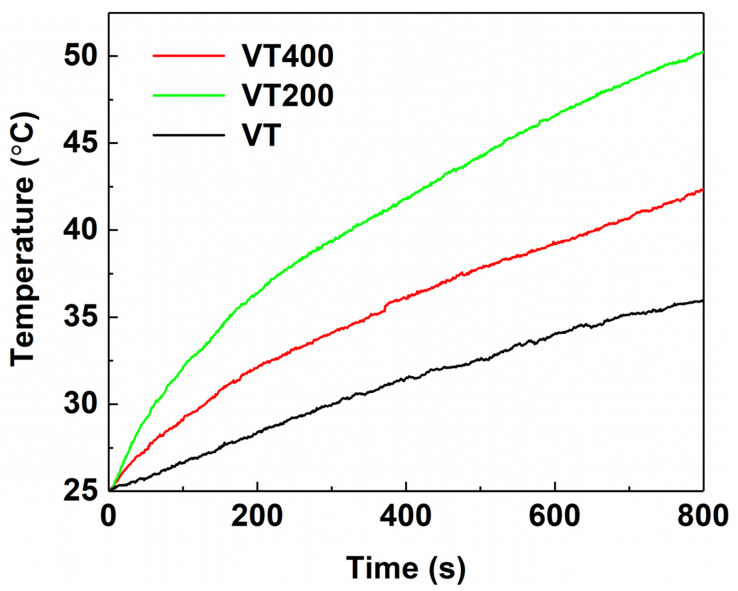
Heating curves of VT, VT200, and VT400 coatings in PBS state at 0.5 W/cm^2^.

The VT600 sample was shed from the Ti surface as a result of pulverization under a higher temperature of 600 °C. The VT600 sample lost the typical porous structure of the plasma spraying coating, which is beneficial for adhering and living. So, the VT600 sample was not discussed in the following studies. The photothermal conversion properties of tantalum coatings of VT, VT200, and VT400 were investigated using an 808 nm laser (intensity: 0.5 Wcm^−2^) in PBS solutions. The temperatures of VT, VT200, and VT400 increase with the increase in irradiation time. Compared with VT, VT200 and VT400 show a relatively rapid rate of increase. As shown in Figure 6, after 600 s, the temperature increases from 25 °C to 32.8 °C, 37.2 °C, and 47 °C for VT, VT400, and VT200, respectively.

### 3.3. Photothermal Effect of Tantalum Coatings for Killing Bone-Tumor Cells

**Figure 7:**
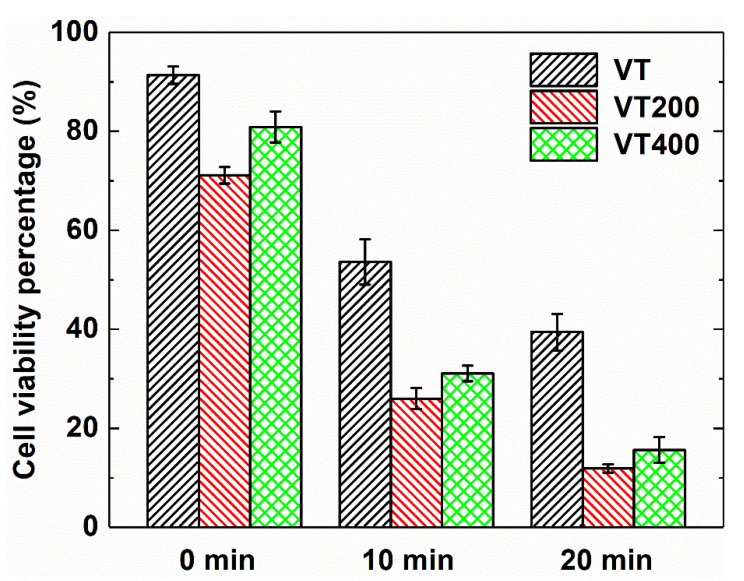
Cell viability percentage of MG-63 cells at different times after photothermal treatment on VT, VT200, and VT400 coating surfaces at 0.5 W/cm^2^.

To investigate the photothermal effect of thermal oxidation treatment at different temperatures on the tantalum coatings, we incubated MG-63 cells with PBS dispersions and then tested their viability on VT, VT200, and VT400 with 808 nm laser irradiation (Figure 7. It can be seen that the viabilities of the MG-63 cells in the coatings are inclined to reduce drastically after irradiation for 10 min. The longer the photothermal treatment time, the weaker the cell activity. As shown in the statistical results of Figure 7, the cell activities decline when the irradiation time increases. VT200 shows the lowest cell activity, which indicates the best photothermal ablation effect in vitro.

**Figure 8:**
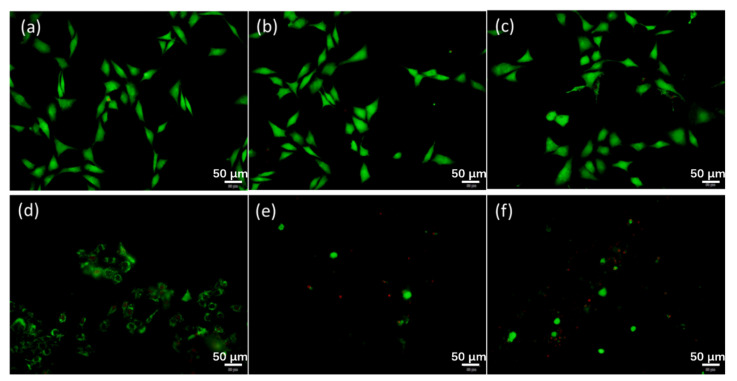
Confocal images of cells on VT, VT200, and VT400 before (**a**–**c** respectively) and after (**d**–**f** respectively) irradiation.

Figure 8 illustrates the confocal images of the cells on VT, VT200, and VT400 before and after irradiation. A large number of living cells showing green fluorescence can be observed on the tantalum coatings before irradiation. After laser irradiation, the number of living cells decreases significantly, especially those on VT200 and VT400, as shown in Figure 8e,f. The tendency towards photothermal effects of different tantalum coatings is in agreement with the results in the CCK-8 assay of cell ablation.

## 4. Conclusions

In summary, we prepared tantalum coatings using a plasma spraying method and thermal treatment. The Ta coating showed partial oxidation after 1 h of heat treatment at low temperatures (200 °C and 400 °C), and the formation of oxygen vacancies narrows the band gap. The Ta coating was completely oxidized after 1 h of heat treatment at a higher temperature (600 °C), and the porous structure of the coating disappeared. Thermal oxidation treatment further enhanced the chemical stability of the coating, and the corrosion resistance increased with the oxidation degree. The photothermal conversion effect of the Ta coating in vitro decreased with the increase in oxygen content. The Ta coating at lower heat treatment temperatures (200 °C and 400 °C) showed better photothermal therapy abilities.

## Data Availability

Not applicable.
